# NOS1AP Interacts with α-Synuclein and Aggregates in Yeast and Mammalian Cells

**DOI:** 10.3390/ijms23169102

**Published:** 2022-08-14

**Authors:** Anton B. Matiiv, Svetlana E. Moskalenko, Olga S. Sergeeva, Galina A. Zhouravleva, Stanislav A. Bondarev

**Affiliations:** 1 Department of Genetics and Biotechnology, St. Petersburg State University, 199034 St. Petersburg, Russia; 2 St. Petersburg Branch, Vavilov Institute of General Genetics, Russian Academy of Sciences, 199034 St. Petersburg, Russia; 3 Laboratory of Amyloid Biology, St. Petersburg State University, 199034 St. Petersburg, Russia

**Keywords:** NOS1AP, CAPON, α-synuclein, protein aggregation, schizophrenia, synucleinopathies

## Abstract

The *NOS1AP* gene encodes a cytosolic protein that binds to the signaling cascade component neuronal nitric oxide synthase (nNOS). It is associated with many different disorders, such as schizophrenia, post-traumatic stress disorder, autism, cardiovascular disorders, and breast cancer. The NOS1AP (also known as CAPON) protein mediates signaling within a complex which includes the NMDA receptor, PSD-95, and nNOS. This adapter protein is involved in neuronal nitric oxide (NO) synthesis regulation via its association with nNOS (NOS1). Our bioinformatics analysis revealed NOS1AP as an aggregation-prone protein, interacting with α-synuclein. Further investigation showed that NOS1AP forms detergent-resistant non-amyloid aggregates when overproduced. Overexpression of *NOS1AP* was found in rat models for nervous system injury as well as in schizophrenia patients. Thus, we can assume for the first time that the molecular mechanisms underlying these disorders include misfolding and aggregation of NOS1AP. We show that NOS1AP interacts with α-synuclein, allowing us to suggest that this protein may be implicated in the development of synucleinopathies and that its aggregation may explain the relationship between Parkinson’s disease and schizophrenia.

## 1. Introduction

Development of diverse neurodegenerative diseases is associated with the appearance of protein aggregates, which usually possess amyloid properties. Amyloids are protein aggregates with a cross-β structure [1,2,3,4]. The discovery of protein co-aggregation with amyloids has revealed the existence of a new and understudied type of intermolecular interactions. It is now becoming more and more obvious that such interactions might play an important role in the pathogenesis of human and animal amyloidosis. Numerous examples of protein co-aggregation are associated with different amyloidosis; for instance, co-aggregation of the following proteins has been shown: amyloid-β (Aβ) and α-synuclein (αSyn), Aβ and amylin, Aβ and PrP, etc. (for further review, see [5]).

Parkinson’s disease is the second most common neurodegenerative disease. It is characterized by abnormal accumulation and aggregation of α-synuclein in the Lewy bodies and Lewy neurites [6]. There is evidence that α-synuclein and amylin cross-seed amyloid fibril formation and that they co-aggregate and form hybrid amyloid fibrils [7]. Despite examples of α-synuclein co-aggregation with other amyloids [5], there are data on its co-aggregation with non-amyloid components as well. Lewy body-like structures and co-aggregates of α-synuclein and Munc18-1 with substitution C180Y were observed in PC12 cells and hippocampal neurons [8]. Thus investigation of α-synuclein co-aggregation with other proteins can help to understand the mechanisms of Parkinson’s disease and develop new therapies. For this purpose, we performed wide-scale bioinformatic screening for aggregation-prone proteins interacting with α-synuclein, and NOS1AP was revealed in this analysis.

The *NOS1AP* (*CAPON*) gene encodes a cytosolic protein that binds neuronal nitric oxide (NO) synthase (nNOS or NOS1) and regulates its activity [9]. NO is an important messenger molecule implicated in different signaling processes. *NOS1AP* is expressed in different tissues: heart, pancreas, skeletal muscles, and nervous system. NOS1AP competes with PSD-93/95 for binding of nNOS. An excess of NOS1AP can inhibit the interaction of PSD-93/95 and nNOS, and thus affect the activation of NO synthase through the N-methyl-D-aspartate (NMDA) receptors in response to Ca${}^{2+}$ influx. NOS1AP also plays a role in the mitogen-activated protein kinase signaling cascade and Hippo signaling pathway through interaction with Dexras 1 and Scribble, respectively (for review see [10,11]).

The *NOS1AP* gene is associated with neurological and psychiatric diseases. The mRNA and protein levels of NOS1AP were shown to be elevated in rat models for nervous system injury, suggesting that this phenomenon was supposed to cause neuron death [12,13]. SNPs in *NOS1AP* are associated with schizophrenia (SCZ) [14,15,16]. The expression of the short *NOS1AP* isoform was elevated in the postmortem brain samples of patients with SCZ [17]. The protein level of different NOS1AP isoforms was also increased in Brodmann area 46 of the frontal cortex in patients diagnosed with SCZ [18]. Overexpression of *NOS1AP* in the hippocampus promotes the development of endophenotypes related to mental disorders [10]. In this work, we investigated the ability of the NOS1AP protein to aggregate in different cells (bacterial, yeast, and mammalian) under overproduction and to co-aggregate with α-synuclein.

## 2. Results

### 2.1. NOS1AP Is a Potential Aggregation-Prone Protein Interacting with α-Synuclein

**Figure 1:**
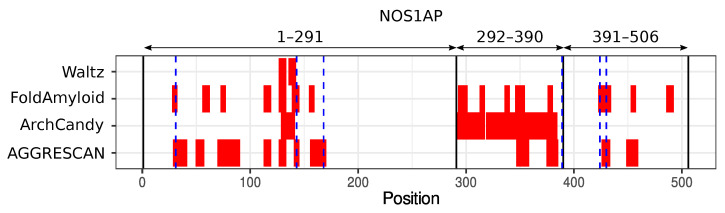
Aggregation-prone regions in NOS1AP. Red rectangles correspond to the potentially amyloidogenic regions. Vertical black lines mark the three regions analyzed in this work. Blue dashed lines designate the positions of cysteines.

NOS1AP was revealed in our bioinformatic screening for amyloidogenic proteins interacting with α-synuclein. We analyzed a set of human proteins which physically interact with α-synuclein according to the BioGRID database [19]. We used the ArchCandy program [20] for the screening. The unique feature of this tool is its ability to predict special structural motifs, called β-arches, which are found in almost all amyloids [20,21]. ArchCandy is reported to be the most accurate tool for modeling amyloid properties [20,22,23]. In addition, we used the IUPred tool [24,25] to find unstructured regions, which are more prone to aggregation than structured regions. A protein was considered as a potential amyloid if it had an unstructured fragment containing at least one β-arch. We analyzed 195 proteins that physically interact with α-synuclein, and found 27 potential amyloids (Figure S1). This work was dedicated to the NOS1AP because it physically interacts with two well-known human amyloids, Aβ and α-synuclein, according to the BioGRID database [19]. For further analysis, we decided to split the protein into three fragments: the most amyloidogenic central part (292–390), and the corresponding N- (1–291) and C-terminal (391–506) fragments preceding and following it, respectively (Figure 1).

We analyzed the amino acid sequence of the NOS1AP protein for potential amyloidogenic regions with other algorithms for modeling of amyloidogenic properties such as Waltz [26], AGGRESCAN [27], and FoldAmyloid [28]. All tools predicted amyloidogenic sequences in the N-terminal fragments (1–291). The central part (292–390) of NOS1AP and its C-terminal region (391–506) are amyloidogenic, according to Waltz and FoldAmyloid (Figure 1).

### 2.2. NOS1AP Does Not Demonstrate Amyloid Properties in C-DAG

**Figure 2:**
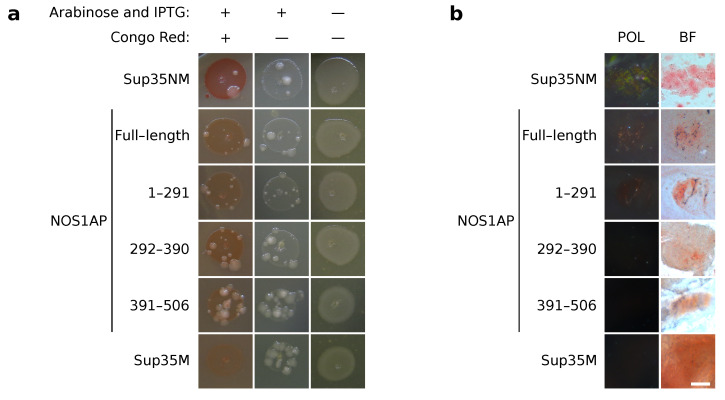
NOS1AP does not form amyloid aggregates in the C-DAG system. (**a**). Bacterial cells, overproduced NOS1AP, or its fragments possess the same color as negative control (Sup35M). The presence of Congo Red and inductors (arabinose and IPTG) are marked above the photographs. (**b**) The bacterial cells from Congo Red-containing media overproduced NOS1AP or its fragments do not demonstrate apple-green birefringence in polarized light. The microphotographs were obtained under transmitted (BF) and cross-polarized (POL) light. Scale bar equals 25 µm. Plasmids encoding Sup35NM and Sup35M were used as positive and negative controls, respectively.

Distinct microbial model systems were developed for express analysis of the protein aggregation [29,30,31]. The bacterial C-DAG system allows analysis of investigated protein aggregates via staining with Congo Red [31]. This feature, accompanied with apple-green birefringence, is considered one of the key pieces of evidence for amyloid properties [1,2,3,4]. In C-DAG, the protein of interest fused with CsgA signal peptide is overproduced in *E. coli* cells. If the protein is amyloid, it forms aggregates on the cell surface that lead to a red color of the colonies on media containing Congo Red, and the same cells demonstrate apple-green birefringence in cross-polarized light [31]. We analyzed the properties of NOS1AP in C-DAG. Full-length protein and all its fragments were indistinguishable from the negative non-amyloid control (Sup35M). Bacteria overproducing these proteins were lightly colored on the Congo Red-containing media (Figure 2a), and did not demonstrate apple-green birefringence (Figure 2b). The positive control, Sup35NM, demonstrated opposite results in both tests (Figure 2). Thus, we are able to conclude that NOS1AP does not form amyloid aggregates in the C-DAG system.

### 2.3. NOS1AP Is Able to Aggregate When Overproduced in Eukaryotic Cells

**Figure 3:**
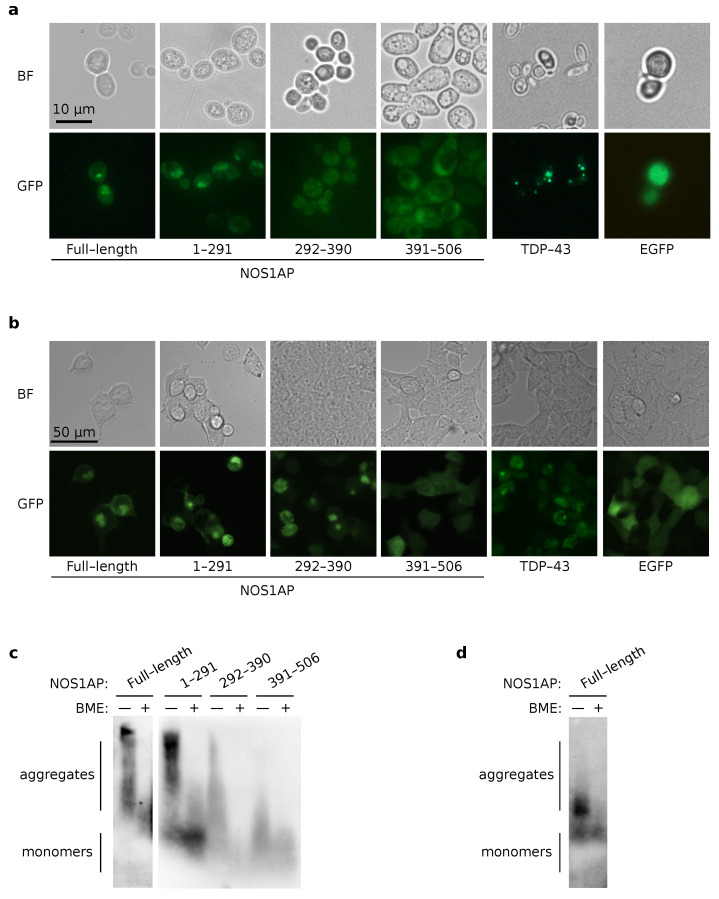
NOS1AP aggregates in yeasts and the HEK293T cell line. (**a**) Microphotographs of yeast cells (2-74-D694) overproducing EGFP-NOS1AP or its fragments. BF—bright field; GFP—fluorescent signal of the protein. (**b**) Microphotographs of HEK293T cells overproducing EGFP-NOS1AP or its fragments. (**c**) Results of SDD-AGE with protein lysates of HEK293T cells overproducing NOS1AP or its fragments. Anti-NOS1AP and anti-Tag(CGY)FP antibodies were used for detection of full-length NOS1AP and its fragments, respectively. (**d**) Results of SDD-AGE with protein lysates of yeast cells overproducing NOS1AP. Anti-Tag(CGY)FP antibodies were used. “+” and “–” mark sample buffers with or without BME, respectively.

Then, we assessed the ability of NOS1AP to aggregate in the eukaryotic model systems, first of all in yeasts. The yeast *S. cerevisiae* strain 2-74-D694 was transformed with plasmids for overproduction of NOS1AP or its fragments fused with EGFP. The analogous plasmids coding TDP-43 (a well-known human amyloid protein; for review, see [1]) and EGFP were used as positive and negative controls, respectively. Transformants were further analyzed by fluorescence microscopy. We observed the bright foci only for the full-length protein and its N-terminal fragment (1-291) (Figure 3a). Appearance of such foci is a characteristic feature of overproduction of aggregation-prone proteins in the yeasts [32,33]. We did not find any aggregates for the fragment 292-390 with this approach (Figure 3b). In addition, we found that overproduction of C-terminal NOS1AP fragments affected cell morphology (Figure 3a). *S. cerevisiae* possesses at least two proteins with PDZ domains (HtrA-like protease and the Nas2 protein, which is involved in assembly of the base subcomplex of the 19S proteasomal regulatory particle) [34]. We can speculate that an excess of NOS1AP fragments containing the PDZ-binding motif may disturb the interaction between yeast proteins containing PDZ domains and their essential partners, and thus lead to morphological changes. In HEK293T3 cells, we observed fluorescent foci with overproduction of both the full-length NOS1AP protein and its fragments 1-291 and 292-390 (Figure 3b).

To investigate NOS1AP aggregation in mammalian cells, the HEK293T cells were transfected with plasmids for overproduction of the full-length protein or its fragments fused with EGFP. After 24 h of incubation, cells were analyzed with fluorescence microscopy. The local accumulation of the full-length protein and its fragments 1–291 and 292–390 were detected in different parts of cells (Figure 3b). Simultaneously, NOS1AP 391–506 was diffusely distributed. Proteins TDP-43 and EGFP were used as positive and negative controls, respectively.

Resistance to detergent treatment is a feature of both amyloid and amyloid-like aggregates (for review see [1]). SDD-AGE has previously been proposed to analyze high molecular weight detergent-resistant protein aggregates [35]. Such large complexes can enter into the agarose gel used in this approach, and can subsequently be detected by western blot hybridization. We found high molecular weight SDS-resistant aggregates of NOS1AP and its fragments 1–291 and 292–390 in the HEK293T cells using this technique [35]. Interestingly, these complexes are stabilized by disulfide bonds because of the addition of β-mercaptoethanol (BME) dissolved aggregates (Figure 3b). Surprisingly, we also detected small aggregates of NOS1AP 391–506 (Figure 3c) which were not detected by fluorescence microscopy (Figure 3a). NOS1AP in yeast cells were represented by dimers and small oligomers, which were resistant to SDS-treatment (Figure 3d). It seems that all aggregates of NOS1AP fragments demonstrate the same sensitivity to BME. This coincides with the distribution of cysteines across the protein; all analyzed fragments contain such residues (Figure 1). Taken together, our results demonstrate that NOS1AP can form SDS-resistant aggregates when overproduced.

### 2.4. NOS1AP Forms Non-Amyloid Aggregates In Vitro

**Figure 4:**
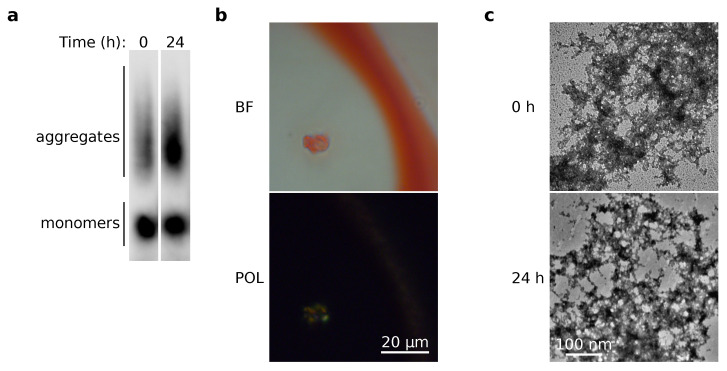
NOS1AP forms detergent resistant but non-amyloid aggregates in vitro. (**a**). Results of SDD-AGE for the NOS1AP samples. Anti-His antibodies were used for detection. (**b**). The microphotograph of the protein sample stained by Congo Red was obtained under transmitted (BF) and cross-polarized (POL) light. (**c**). TEM microphotographs of NOS1AP aggregates.

To further investigate of the biochemical properties of NOS1AP complexes, we obtained aggregates of the protein in vitro. The protein was purified in denaturing conditions (in the presence of 8M urea), concentrated, and then diluted into a buffer without detergent. NOS1AP formed SDS-resistant aggregates immediately after dilution; such aggregates are stable within 24 h of incubation at 37 °C (Figure 4a). Congo Red staining revealed that these complexes are non-amyloid. We did not observe characteristic apple-green birefringence under cross-polarized light (Figure 4b). Only a few slightly glowing particles were found across the samples. This result coincided with electron microscopy data; the approach we used revealed that NOS1AP formed amorphous but not fibrillar aggregates (Figure 4b).

### 2.5. NOS1AP Interacts with α-Synuclein in Mammalian and Yeast Cells

**Figure 5:**
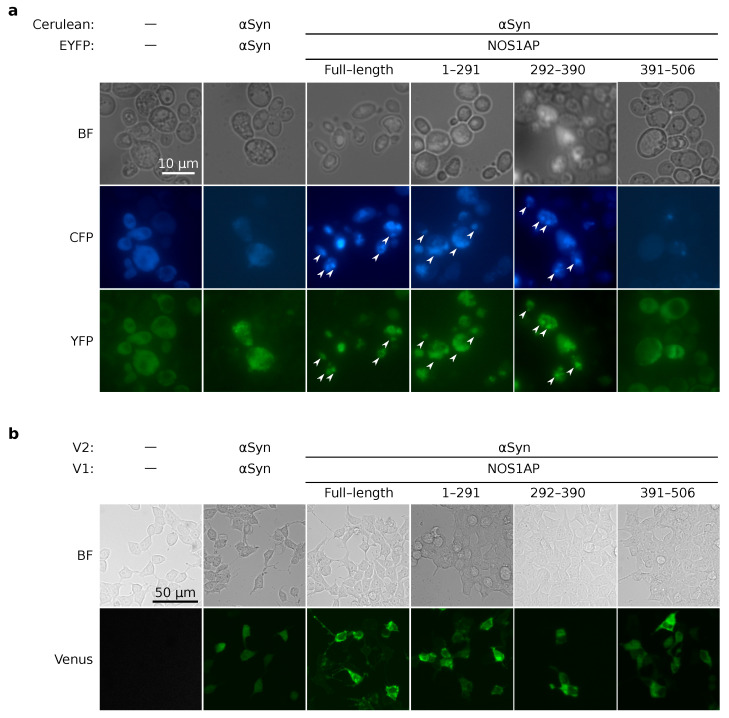
NOS1AP interacts with α-synuclein in yeast and mammalian cells. (**a**). Microphotographs of yeast cells overproducing EYFP-NOS1AP (full-length or truncated) and Cerulean-αSyn. Arrowheads mark examples of co-localisation of bright foci formed by different proteins. (**b**) Microphotographs of HEK293T cells overproducing proteins of interest fused with N- and C-terminal fragments of Venus protein, designated V1 and V2, respectively.

Interaction of NOS1AP and α-synuclein was detected by proteome-wide screening [36]. We performed target verification of this fact with two eukaryotic model systems. The yeast strain 2-74-D694 was co-transformed with centromeric plasmids for overproduction of NOS1AP or its fragments fused with EYFP and plasmid for overproduction of Cerulean-αSyn. The use of different fluorescent proteins allowed us to monitor the localisation of two proteins in the same cells. The transformants were analyzed with fluorescence microscopy. We observed colocalization of bright foci formed by α-synuclein and NOS1AP (full-length, 1–291 and 292–390). It should be noted that in the absence of overproduction of α-synuclein, NOS1AP 292–390 is scattered in the cytoplasm (compare Figure 3a and Figure 5a). At the same time, NOS1AP 391–506 as well as the fluorescent proteins (Cerulean and EYFP) were diffusely distributed in the cytoplasm of cells (Figure 5a). These results allow us to suppose that NOS1AP and α-synuclein interact with each other in the yeast cells, and that even more can co-aggregate.

The interaction of NOS1AP and α-synuclein in mammalian cells was assessed with the bimolecular fluorescence complementation assay. We used a two-plasmid system including the pDEST-V1-ORF and pDEST-V2-ORF vectors for overproduction of the investigated proteins fused with fragments of Venus protein (V1 and V2), which are non-fluorescent. Physical interaction of the analyzed proteins leads to reassociation of V1 and V2 and to the recovery of fluorescent properties. Cells co-transfected with pDEST-V1-NOS1AP and pDEST-V2-NOS1AP (data not shown) or pDEST-V1-αSyn and pDEST-V2-αSyn (Figure 5b) demonstrated fluorescence of Venus protein. Both combinations represented positive controls in this experiment. At the same time, we observed no fluorescence in cells co-transfected with empty vectors, which produced only V1 and V2 fragments (negative control, Figure 5b). These two facts proved the reliability of this approach. We then demonstrated that NOS1AP and all its fragments interact with α-synuclein; we detected the fluorescence for all combinations of plasmids (Figure 5b). We obtained the same results for the reciprocal combination of Venus fragments, V1-αSyn and V2-NOS1AP (data not shown). It should be mentioned that α-synuclein itself did not form bright fluorescent foci in this experiment (Figure 5). However, we observed small bright foci when NOS1AP (full-length or fragment 1–291) and α-synuclein were overproduced (Figure 5b). This allows us to suppose that the NOS1AP 1–291 fragment is responsible for co-aggregation with α-synuclein in mammalian cells.

## 3. Discussion

### 3.1. NOS1AP Can Form Non-Amyloid Aggregates When Overproduced

The search for new potentially amyloidogenic or aggregation-prone proteins is an important task in both biology and medicine. Amyloids are associated with serious diseases such as Alzheimer’s disease (AD), Parkinson’s disease (PD), amyotrophic lateral sclerosis, type 2 diabetes, cerebral autosomal dominant arteriopathy, renal disease, fibronectin glomerulopathy, and many others [37]. Most of these diseases are incurable and even fatal to humans. The investigation of new examples of protein aggregation and co-aggregation is an important fundamental task in biology, and holds out the possibility of discovering new targets for the treatment of corresponding diseases. The lack of detailed understanding of the molecular mechanisms of protein aggregates’ toxicity is one of the key reasons for the lack of effective therapies. In this work, we performed a bioinformatic search of amyloidogenic proteins among proteins physically interacting with α-synuclein and found several candidates, including NOS1AP (Figure 1 and Figure S1).

We demonstrated for the first time that the NOS1AP protein can aggregate in eukaryotic model systems under conditions of overproduction (Figure 3). We found that the full-length protein and N-terminal fragment 1–291 can form aggregates when overproduced both in yeast cells and in human cells. However, aggregation of the 292–390 fragment was observed by fluorescence only in HEK293T cells. The C-terminal part of the protein (391–506) did not form large aggregates (Figure 3).

The aggregates of the full-length NOS1AP protein formed in HEK293T cells, as well as its fragments 1–291 and 292–390, are SDS-resistant (Figure 3). These complexes are likely stabilized by disulfide bonds, as they are sensitive to BME (Figure 3c) [38]. The full-length NOS1AP protein formed SDS-resistant, BME-sensitive aggregates in yeast cells (Figure 3). Thus, we could conclude that the aggregation-prone properties of the protein are not restricted by one model system. Previously, at least two examples of protein aggregates stabilized by disulfide bonds have been described: amyloid of β2-microglobulin [39] and NUP58 [40].

NOS1AP formed amorphous aggregates in vitro that did not show apple-green birefringence in cross-polarized light after Congo Red staining (Figure 4). The last feature is one of the essential properties of amyloids. These observations coincide with the C-DAG results (Figure 2). Thus, we can conclude that the analyzed protein tends to assemble into non-amyloid amorphous aggregates in near-physiological conditions. However, we cannot exclude the possibility that, under certain conditions, NOS1AP can also form amyloids.

The NOS1AP protein contains two potentially amyloidogenic regions predicted by the ArchCandy program: fragment 1–291 and 292–390 (Figure 1). It can be noted that the analysis of NOS1AP aggregation in eukaryotic cells is mainly consistent with these predictions; regions with β-arches are responsible for aggregation. Other programs used provided less accurate results; Walts found only one amyloidogenic region, while AGGRESCAN and FoldAmyloid predicted the C-terminal region as aggregation-prone.

### 3.2. Potential Role of NOS1AP Aggregation in Schizophrenia Development

Increased expression and protein overproduction of NOS1AP have been shown in the brain samples of patients with mental disorders, especially SCZ [11]. An analogous result was obtained in another work for a short transcript of *NOS1AP* [17]. This isoform includes sequence coding fragment 292–390, which can aggregate (Figure 1), and a PDZ-binding motif. It has been suggested that binding of overproduced monomeric NOS1AP and nNOS possibly results in a reduction of functional NMDA receptor/nNOS complexes, leading to inactivation of NMDA receptors (gated calcium influx and a fermentative inactive nNOS) [41].

**Figure 6:**
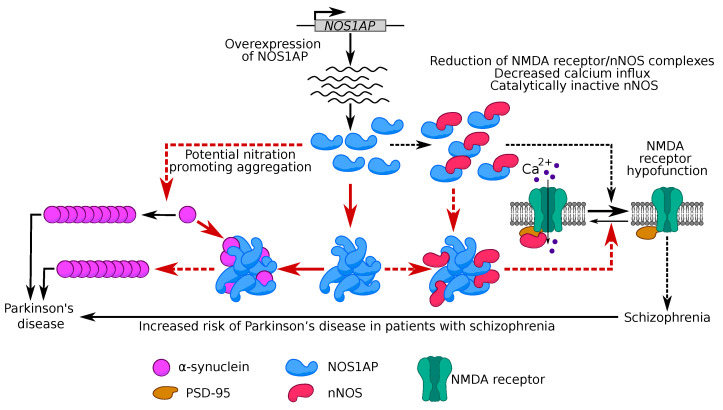
Possible roles of NOS1AP aggregation in pathogenesis of Parkinson’s disease and schizophrenia. Solid lines correspond to experimental results and dashed lines mark hypothetical connections. The red arrows indicate the processes and the hypotheses that we put forward based on our results.

We have shown that NOS1AP and its fragments 1–291 and 292–390 are able to form stable aggregates (Figure 3). The inactivation of different proteins in aggregates of their functional partners has been shown in many examples (for further review, see [5]). This allows us to assume that NOS1AP aggregates are able to titrate nNOS, which prevents the realization of its functional role in the cell and subsequent inactivation of NMDA receptors. It has been supposed that a decrease in the activity of these receptors is linked to the development of SCZ [42]. Thus, our data allow us to propose a new molecular mechanism, based on NOS1AP aggregation, underlying this hypothesis (Figure 6).

### 3.3. Potential Role of NOS1AP and α-Synuclein Co-Aggregation in Development of Parkinson’s Disease

While the interaction of the NOS1AP protein with α-synuclein has previously been shown by high-throughput proteomic screening [36], corresponding target verification was absent. Thus, we had the opportunity to investigate for the first time the physical interaction of NOS1AP with α-synuclein by in-depth analysis and to search for the fragments required for formation of this protein complex. Our results for HEK293T cells demonstrated that binding of α-synuclein and NOS1AP is non-specific (Figure 5b). In yeast cells, we were able to detect the co-localization of these two proteins in aggregates and suppose the absence of co-aggregation between α-synuclein and NOS1AP (391–506) (Figure 5a). This discrepancy between two systems may be explained as follows. The fragment, including a functional PDZ-binding motif, interacts with yeast proteins with PDZ domains, thus affecting α-synuclein binding. Furthermore, the absence of a specific binding site between proteins allows us to suppose that we observed sequestration of α-synuclein in NOS1AP aggregates. We observed that α-synuclein co-aggregates with NOS1AP and its N-terminus (1–291) in HEK293T cells (Figure 5b), but is diffusely distributed when overproduced alone. This supports the hypothesis mentioned above. At the same time, aggregation of NOS1AP itself seems to be independent of α-synuclein, because we observed it both in yeast cells (Figure 3) and in vitro (Figure 4).

As mentioned above, the NOS1AP protein is associated with psychiatric disorders, mainly SCZ [11]. Previously, cases of coexistence of PD, well-known synucleinopathy, and SCZ have been described [43]. In addition, there is evidence that patients with SCZ spectrum disorders have an increased risk of developing PD [44]. Thus, it is possible to assume that NOS1AP is implicated in the development of synucleinopathies. Our data on the interaction and co-aggregation of these two proteins suggest a molecular mechanism underlying the coexistence of the two pathologies. The increase of local concentration of α-synuclein in NOS1AP aggregates may promote its aggregation and development of synucleinopathies (Figure 6).

From another point of view, a mechanism based on modification of α-synuclein can also explain the relationship between PD and SCZ. In mice, *NOS1AP* overexpression induced significantly higher levels of phosphorylated, oligomerized, and insoluble tau, as well as an increase in nitration of tau at Tyr29 [45]. Nitration is one of the possible oxidative mechanisms leading to the formation of α-synuclein oligomers via dityrosine crosslinking [46]. Nitrided α-synuclein has been found in Lewy bodies in the brain samples of patients with PD [47,48,49]. For instance, soluble α-synuclein nitrated in Tyr39 is not efficiently bound by proteases, which leads to a decrease in the rate of protein degradation, accumulation, and formation of fibrils [50]. Thus, we can suppose that overproduction of NOS1AP can also lead to α-synuclein nitration and affect its aggregation (Figure 6).

## 4. Methods and Materials

### 4.1. Bioinformatic Analysis

The set of proteins physically interacting with α-synuclein was obtained from the BioGRID database [19]. ArchCandy was used to predict the amyloidogenic properties of proteins (threshold value 0.575) [20]. The IUPred program (with the option “long”) was used to find unstructured regions [24,25]. The protein fragments with an IUPred score more than 0.3 were considered as unstructured. A protein or its part was considered amyloidogenic if at least one β-arch (based on ArchCandy predictions) with a score above the threshold was located in an unstructured region.

### 4.2. Plasmid Construction

Plasmids pDONR221-NOS1AP-1-291, pDONR221-NOS1AP-292-390, and pDONR221-NOS1AP-391-506 were obtained by Gateway cloning. The PCR products of corresponding sequences of *NOS1AP* flanked with *att*B sites were inserted into pDONR221-ccdB (Thermo Scientific, Waltham, MA, USA), 12536017) by BP reaction (BP Clonase™ II Enzyme mix, Thermo Scientific). This reaction leads to recombination between *att*B and *att*P sites in the PCR product and donor vector, respectively. The plasmid pDONR221-NOS1AP (Thermo Scientific Ultimate ORF Clone IOH42227) was used as a template. All primers used are listed in Table 1. These entry clones were used for construction of expression vectors. The corresponding coding sequences were inserted into different donor vectors listed in Table 2 by LR reaction (LR Clonase™ II Enzyme mix, Thermo Scientific), leading to a recombination between *att*L and *att*R sites in the entry clone (for instance pDONR221-NOS1AP) and destination vector, respectively. The vector pDONR221-TARDBP (Thermo Scientific Ultimate ORF Clone IOH45677) was used to construct of plasmids for overproduction of TDP-43. All yeast vectors (pAG416GPD-EGFP-ccdB, pAG416GPD-EYFP-ccdB, and pAG415GPD-Cerulean-ccdB) were low copy number centromeric plasmids.

Standard microbiological approaches and media were used for all manipulations with bacteria [38]. The *Escherichia coli* strains TOP10 (Invitrogen) and DH5α [51] were used for cloning. The DB3.1 strain (Thermo Scientific) was used as a host for plasmids with *ccdB* cassette.

**Table 2:** Donor vectors.

Vector	Signal Peptide, Fluorescent Protein, Tag, and Promoter	Experiment	Reference
pVSGW-ccdB	CsgA signal peptide on the N-termini of a protein, *BAD* promoter	C-DAG system	[40]
pgLAP1	EGFP on the N-termini of a protein, CMV promoter	Analysis of protein localization in mammalian cells	A gift from Peter Jackson (Addgene plasmid #19702 http://n2t.net/addgene:19702 accessed on 13 July 2022)
pDEST-V1-ORF	N-terminal fusion protein with Venus fluorescent protein fragment 1 (V1), CMV promoter	Analysis of protein-protein interaction in mammalian cells	A gift from Darren Saunders (Addgene plasmid #73635 http://n2t.net/addgene:73635 accessed on 13 July 2022) [52]
pDEST-V2-ORF	N-terminal fusion protein with Venus fluorescent protein fragment 2 (V2), CMV promoter	Analysis of protein-protein interaction in mammalian cells	A gift from Darren Saunders (Addgene plasmid #73636 http://n2t.net/addgene:73636 accessed on 13 July 2022) [52]
pAG416GPD-EGFP-ccdB	EGFP on the N-termini of a protein, *GAP* promoter	Analysis of protein localization in yeast cells	A gift from Susan Lindquist (Addgene plasmid #14316 http://n2t.net/addgene:14316 accessed on 13 July 2022)
pAG416GPD-EYFP-ccdB	EYFP on the N-termini of a protein, *GAP* promoter	Analysis of protein localization in yeast cells	A gift from Susan Lindquist (Addgene plasmid #14340 http://n2t.net/addgene:14340 accessed on 13 July 2022)
pAG415GPD-Cerulean-ccdB	Cerulean on the N-termini of a protein, *GAP* promoter	Analysis of protein localization in yeast cells	A gift from Susan Lindquist (Addgene plasmid #14410 http://n2t.net/addgene:14410 accessed on 13 July 2022)
pDest-527	His6 tag on the N-termini of a protein, T7 promoter	Protein purification	A gift from Dominic Esposito (Addgene plasmid #11518 http://n2t.net/addgene:11518 accessed on 13 July 2022)

### 4.3. Yeast Strains, Transformation, and Microscopy

The *Saccharomyces cerevisiae* 2-74-D694 strain (*MATa ade1-14(UGA) trp1-289(UAG) ura3-52 his3-*∆*200 leu2-3,112* [*psi*^–^] [*pin*^–^], a gift from A.G. Matveenko) was used in all experiments. This strain is a derivative of 74-D694 [53] obtained by several passages on media supplemented with guanidinium chloride for prion elimination. The transformation of yeast cells was performed according to the published protocol [54] with minor modifications. Cells were cultivated in YEPD or synthetic complete media at 30 °C [55]. For microscopy, cells were grown in liquid media to the logarithmic phase (OD_600_ = 0.6), then gently pelleted (800 g) and resuspended in 50% (*v*/*v*) glycerol. Fluorescence was analyzed using a Zeiss AxioScope A1 wide-field fluorescence microscope. Images were taken with a QIClick-F-CLR-12 (QImaging) or Axiocam 506 color (Zeiss) camera using QCAPTURE PRO 7 or ZEN 3.4 (blue edition) software, respectively.

### 4.4. Curli-Dependent Amyloid Generator (C-DAG)

For the experiments in the C-DAG system, the *E. coli* strain VS39 was transformed with the target plasmids and transformants were selected on LB medium supplemented with ampicillin and chloramphenicol at 37 °C [31]. The overnight bacterial culture was diluted 100-fold and grown for one hour. The obtained cultures were plated on series of media: CR inducing plate (0.2% (*w*/*v*) L-arabinose, 0.1 mM IPTG, 10 µg/mL Congo Red dye, 200 µg/mL ampicillin and 25 µg/mL chloramphenicol), and a control plate containing only antibiotics. Plates were incubated at 26 °C for 5 days. The plasmids pVSGW-Sup35NM, pVSGW-Sup35M [40] were used as positive and negative controls, respectively. Samples for polarization microscopy were prepared as follows: 20 µL of the cell suspension were applied on a slide and dried, then the cells were covered with 5 µL of 50% (*v*/*v*) glycerol and analyzed with an inverted Leica DMI6000 microscope.

### 4.5. Cell Transfection and Microscopy

The HEK293T cell line was kindly gifted by Natalia Katolikova, a researcher at Institute of Translational Biomedicine of Saint Petersburg State University. Cells were cultured in DMEM media supplemented with 10% (*v*/*v*) fetal bovine serum and with penicillin (100 U/mL) and streptomycin (100 µg/mL). The TurboFect (Thermo Fisher Scientific, R0532, Waltham, MA, USA) reagent was used for transfection according to the manufacturer’s recommendations. Localization of proteins fused with Venus fragments or EGFP were analyzed by fluorescence microscopy (Leica DMI6000 B) 24 h after transfection.

### 4.6. NOS1AP Antibodies

The pDest-527-NOS1AP plasmid and *E. coli* strain BL21(DE3) [56] were used for NOS1AP purification. The protein was purified in denaturing conditions (85 mM Tris-HCl pH 8.0, 8M Urea) with Ni-NTA agarose (Invitrogen). For rabbit immunization, the protein was transferred to the PBS buffer. The specificity of the serum after two rounds of immunization (dilution 1:500) was verified on the recombinant protein and HEK293T cell lysates.

### 4.7. NOS1AP Aggregation, Electron Microscopy, and Congo Red Staining

The purified NOS1AP protein was diluted to a concentration of 0.8 mg/mL in buffer (20 mM Tris-HCl (pH 7.6), 150 mM NaCl) and incubated at 37 °C with stirring. One aliquot was taken immediately after the dilution and another one after 24 h of incubation. These samples were used for further experiments. To prepare specimens for transmission electron microscopy, the protein solution was applied to a copper formvar-coated grid and incubated for 30 s. Then, the grid was washed with water and stained for 30 s with 1% (*w*/*v*) uranyl acetate. Excess dye was removed with water. To prepare specimens for polarization microscopy, the NOS1AP protein solution was applied to a glass slide and stained with Congo Red solution (2.5 mg/mL) for 5 min. Excess dye was removed with ethanol and the sample was dried at room temperature. The sample was then covered with a glycerol solution and a coverslip.

### 4.8. Protein Electrophoresis and Hybridization

Protein extraction from the HEK293T cells was performed as follows. Pelleted cells were resuspended in RIPA buffer (150 mM NaCl, 1% (*v*/*v*) Triton X-100, 0.5% (*w*/*v*) sodium deoxycholate, 0.1% (*w*/*v*) SDS, 50 mM Tris-HCl (pH 8.0), 2 mM PMSF, 2 µL leupeptin (10 µg/mL), 20% (*v*/*v*) protease inhibitor cocktail (Sigma, P8340)) and incubated on ice for 30 min. The suspension was sonicated with a Bandelin sonoplus device (Teopal) for 10 s at 50% device power. Cell debris was pelleted by centrifugation at 800× *g* for 10 min at 4 °C. Yeast cells were grown on a selective medium to OD_600_ = 0.4 and collected by centrifugation. Protein extraction was performed according to the published protocol [57] with the exception of a modified lysis buffer (100 mM Tris-HCl (pH 7.6), 2 mM PMSF, 10 mM BME, 4% (*v*/*v*) protease inhibitors (Sigma, P8215)). Obtained protein lysate was used for semi-denaturing detergent–agarose gel electrophoresis (SDD-AGE). SDD-AGE was performed according to the published protocol with minor modifications [35,58]. Gels with 2% (*w*/*v*) agarose were run at 30 V for 200–240 min in all experiments. Anti-NOS1AP antibodies obtained in our laboratory, commercial anti-Tag(CGY)FP (Evrogen AB121), and anti-His (GE Healthcare) antibodies were used to detect NOS1AP protein and its fragments fused with EGFP or His6-tag, respectively.

## 5. Conclusions

The NOS1AP protein, which is linked to schizophrenia development, can form detergent-resistant non-amyloid aggregates when overproduced. This discovery reveals a new molecular mechanism, potentially explaining the inactivation of NMDA receptors in this pathology. The co-aggregation of NOS1AP and α-synuclein provides an explanation of the described linkage between PD and SCZ; however, the molecular mechanism of this relationship remains in question.

## Figures and Tables

**Table 1:** Primers.

Primer	Sequence
NOS1AP-F-292-390	GGGGACAAGTTTGTACAAAAAAGCAGGCTACCAGATGCAGCTCC
R_NOS1AP(HindIII)_attB2	GGGGACCACTTTGTACAAGAAAGCTGGGTAAGCTTCTAGTCACAGAGCACGGGCAG
NOS1AP_1_291_NAR_attB1 forward_primer	GGGGACAAGTTTGTACAAAAAAGCAGGCTTCATGCCTAGCAAAACCAAG
NOS1AP_1_291_NAR_attB2 reverse_primer	GGGGACCACTTTGTACAAGAAAGCTGGGTTCTAGTGGTGAGTGGACAG
NOS1AP_391_506_NAR_attB1 forward_primer	GGGGACAAGTTTGTACAAAAAAGCAGGCTTCCCCACGACCCCTAAGCC
NOS1AP_391_506_ NAR_attB2 reverse_primer	GGGGACCACTTTGTACAAGAAAGCTGGGTTCTACACGGCGATCTCATC

## Data Availability

Not applicable.

## Supplementary Materials

The supporting information can be downloaded at: https://www.mdpi.com/article/10.3390/ijms23169102/s1.

## Abbreviations

The following abbreviations are used in this manuscript:

Aβ	amyloid-β
AD	Alzheimer’s disease
BME	β-mercaptoethanol
C-DAG	curli-dependent amyloid generator
NMDA	N-methyl-D-aspartate
NO	nitric oxide
PD	Parkinson’s disease
SCZ	Schizophrenia
SDD-AGE	semi-denaturing detergent-agarose gel electrophoresis
αSyn	α-synuclein

## References

[1] Matiiv A.B., Trubitsina N.P., Matveenko A.G., Barbitoff Y.A., Zhouravleva G.A., Bondarev S.A. (2020). Amyloid and amyloid-like aggregates: Diversity and the term crisis. Biochemistry.

[2] Sergeeva A.V., Galkin A.P. (2020). Functional amyloids of eukaryotes: Criteria, classification, and biological significance. Curr. Genet..

[3] Nizhnikov A.A., Antonets K.S., Inge-Vechtomov S.G. (2015). Amyloids: From pathogenesis to function. Biochemistry.

[4] Benson M.D., Buxbaum J.N., Eisenberg D.S., Merlini G., Saraiva M.J., Sekijima Y., Sipe J.D., Westermark P. (2020). Amyloid nomenclature 2020: Update and recommendations by the International Society of Amyloidosis (ISA) nomenclature committee. Amyloid.

[5] Bondarev S., Antonets K., Kajava A., Nizhnikov A., Zhouravleva G. (2018). Protein co-aggregation related to amyloids: Methods of investigation, diversity, and classification. Int. J. Mol. Sci..

[6] Gómez-Benito M., Granado N., García-Sanz P., Michel A., Dumoulin M., Moratalla R. (2020). Modeling Parkinson’s Disease With the Alpha-Synuclein Protein. Front. Pharmacol..

[7] Mucibabic M., Steneberg P., Lidh E., Straseviciene J., Ziolkowska A., Dahl U., Lindahl E., Edlund H. (2020). *α*-Synuclein promotes IAPP fibril formation in vitro and *β*-cell amyloid formation in vivo in mice. Sci. Rep..

[8] Chai Y.J., Sierecki E., Tomatis V.M., Gormal R.S., Giles N., Morrow I.C., Xia D., Götz J., Parton R.G., Collins B.M. (2016). Munc18-1 is a molecular chaperone for *α*-synuclein, controlling its self-replicating aggregation. J. Cell Biol..

[9] Jaffrey S.R., Snowman A.M., Eliasson M.J., Cohen N.A., Snyder S.H. (1998). CAPON: A protein associated with neuronal nitric oxide synthase that regulates its interactions with PSD95. Neuron.

[10] Freudenberg F., Alttoa A., Reif A. (2015). Neuronal nitric oxide synthase (NOS1) and its adaptor, NOS1AP, as a genetic risk factors for psychiatric disorders. Genes, Brain Behav..

[11] Wang J., Jin L., Zhu Y., Zhou X., Yu R., Gao S. (2016). Research progress in NOS1AP in neurological and psychiatric diseases. Brain Res. Bull..

[12] Cheng C., Li X., Gao S., Niu S., Chen M., Qin J., Guo Z., Zhao J., Shen A. (2008). Expression of CAPON after spinal cord injury in rats. J. Mol. Neurosci..

[13] Cui Z., Lv Q., Yan M., Cheng C., Guo Z., Yang J., Chen M., Xia Y., Zhang L., Shen A. (2011). Elevated expression of CAPON and neuronal nitric oxide synthase in the sciatic nerve of rats following constriction injury. Vet. J..

[14] Brzustowicz L.M., Simone J., Mohseni P., Hayter J.E., Hodgkinson K.A., Chow E.W., Bassett A.S. (2004). Linkage disequilibrium mapping of schizophrenia susceptibility to the CAPON region of chromosome 1q22. Am. J. Hum. Genet..

[15] Zheng Y., Li H., Qin W., Chen W., Duan Y., Xiao Y., Li C., Zhang J., Li X., Feng G. (2005). Association of the carboxyl-terminal PDZ ligand of neuronal nitric oxide synthase gene with schizophrenia in the Chinese Han population. Biochem. Biophys. Res. Commun..

[16] Miranda A., García J., López C., Gordon D., Palacio C., Restrepo G., Ortiz J., Montoya G., Cardeño C., Calle J. (2006). Putative association of the carboxy-terminal PDZ ligand of neuronal nitric oxide synthase gene (CAPON) with schizophrenia in a Colombian population. Schizophr. Res..

[17] Xu B., Wratten N., Charych E.I., Buyske S., Firestein B.L., Brzustowicz L.M. (2005). Increased expression in dorsolateral prefrontal cortex of CAPON in schizophrenia and bipolar disorder. PLoS Med..

[18] Hadzimichalis N.M., Previtera M.L., Moreau M.P., Li B., Lee G.H., Dulencin A.M., Matteson P.G., Buyske S., Millonig J.H., Brzustowicz L.M. (2010). NOS1AP protein levels are altered in BA46 and cerebellum of patients with schizophrenia. Schizophr. Res..

[19] Oughtred R., Rust J., Chang C., Breitkreutz B.J., Stark C., Willems A., Boucher L., Leung G., Kolas N., Zhang F. (2021). The BioGRID database: A comprehensive biomedical resource of curated protein, genetic, and chemical interactions. Protein Sci. Publ. Protein Soc..

[20] Ahmed A.B., Znassi N., Château M.T., Kajava A.V. (2015). A structure-based approach to predict predisposition to amyloidosis. Alzheimer’S Dement..

[21] Kajava A.V., Baxa U., Steven A.C. (2010). *β* arcades: Recurring motifs in naturally occurring and disease-related amyloid fibrils. Faseb J..

[22] Bondarev S.A., Zhouravleva G.A., Belousov M.V., Kajava A.V. (2015). Structure-based view on [*PSI*^+^] prion properties. Prion.

[23] Roche D.B., Villain E., Kajava A.V. (2017). Usage of a dataset of NMR resolved protein structures to test aggregation versus solubility prediction algorithms. Protein Sci. Publ. Protein Soc..

[24] Dosztányi Z., Csizmók V., Tompa P., Simon I. (2005). The pairwise energy content estimated from amino acid composition discriminates between folded and intrinsically unstructured proteins. J. Mol. Biol..

[25] Dosztanyi Z., Csizmok V., Tompa P., Simon I. (2005). IUPred: Web server for the prediction of intrinsically unstructured regions of proteins based on estimated energy content. Bioinformatics.

[26] Maurer-Stroh S., Debulpaep M., Kuemmerer N., De La Paz M.L., Martins I.C., Reumers J., Morris K.L., Copland A., Serpell L., Serrano L. (2010). Exploring the sequence determinants of amyloid structure using position-specific scoring matrices. Nat. Methods.

[27] Conchillo-Solé O., de Groot N.S., Avilés F.X., Vendrell J., Daura X., Ventura S. (2007). AGGRESCAN: A server for the prediction and evaluation of “hot spots” of aggregation in polypeptides. BMC Bioinform..

[28] Garbuzynskiy S.O., Lobanov M.Y., Galzitskaya O.V. (2009). FoldAmyloid: A method of prediction of amyloidogenic regions from protein sequence. Bioinformatics.

[29] Rubel M.S., Fedotov S.A., Grizel A.V., Sopova J.V., Malikova O.A., Chernoff Y.O., Rubel A.A. (2020). Functional mammalian amyloids and amyloid-like proteins. Life.

[30] Chandramowlishwaran P., Sun M., Casey K.L., Romanyuk A.V., Grizel A.V., Sopova J.V., Rubel A.A., Nussbaum-Krammer C., Vorberg I.M., Chernoff Y.O. (2018). Mammalian amyloidogenic proteins promote prion nucleation in yeast. J. Biol. Chem..

[31] Sivanathan V., Hochschild A. (2013). A bacterial export system for generating extracellular amyloid aggregates. Nat. Protoc..

[32] Alberti S., Halfmann R., King O., Kapila A., Lindquist S. (2009). A systematic survey identifies prions and illuminates sequence features of prionogenic proteins. Cell.

[33] Liebman S.W., Chernoff Y.O. (2012). Prions in yeast. Genetics.

[34] Muley V.Y., Akhter Y., Galande S., Gojobori T. (2019). PDZ domains across the microbial world: Molecular link to the proteases, stress response, and protein synthesis. Genome Biol. Evol..

[35] Kryndushkin D.S., Alexandrov I.M., Ter-Avanesyan M.D., Kushnirov V.V. (2003). Yeast [*PSI*^+^] prion aggregates are formed by small Sup35 polymers fragmented by Hsp104. J. Biol. Chem..

[36] Hein M.Y., Hubner N.C., Poser I., Cox J., Nagaraj N., Toyoda Y., Gak I.A., Weisswange I., Mansfeld J., Buchholz F. (2015). A human interactome in three quantitative dimensions organized by stoichiometries and abundances. Cell.

[37] Chiti F., Dobson C.M. (2017). Protein misfolding, amyloid formation, and human disease: A summary of progress over the last decade. Annu. Rev. Biochem..

[38] Sambrook J., Fritsch E.F., Maniatis T. (1989). Molecular Cloning: A Laboratory Manual.

[39] Liu C., Sawaya M.R., Eisenberg D. (2011). *β*2-microglobulin forms three-dimensional domain-swapped amyloid fibrils with disulfide linkages. Nat. Struct. Mol. Biol..

[40] Danilov L.G., Moskalenko S.E., Matveenko A.G., Sukhanova X.V., Belousov M.V., Zhouravleva G.A., Bondarev S.A. (2021). The human NUP58 nucleoporin can form amyloids in vitro and in vivo. Biomedicines.

[41] Eastwood S.L. (2005). Does the *CAPON* gene confer susceptibility to schizophrenia?. PLoS Med..

[42] Moghaddam B., Javitt D. (2012). From revolution to evolution: The glutamate hypothesis of schizophrenia and its implication for treatment. Neuropsychopharmacology.

[43] Winter C., Juckel G., Plotkin M., Niehaus L., Kupsch A. (2006). Paranoid schizophrenia and idiopathic Parkinson’s disease do coexist: A challenge for clinicians. Psychiatry Clin. Neurosci..

[44] Kuusimäki T., Al-Abdulrasul H., Kurki S., Hietala J., Hartikainen S., Koponen M., Tolppanen A.M., Kaasinen V. (2021). Increased risk of Parkinson’s disease in patients with schizophrenia spectrum disorders. Mov. Disord..

[45] Hashimoto S., Matsuba Y., Kamano N., Mihira N., Sahara N., Takano J., Muramatsu S.i., Saido T.C., Saito T. (2019). Tau binding protein CAPON induces tau aggregation and neurodegeneration. Nat. Commun..

[46] Souza J.M., Giasson B.I., Chen Q., Lee V.M., Ischiropoulos H. (2000). Dityrosine cross-linking promotes formation of stable *α*-synuclein polymers: Implication of nitrative and oxidative stress in the pathogenesis of neurodegenerative synucleinopathies. J. Biol. Chem..

[47] Takahashi T., Yamashita H., Nakamura T., Nagano Y., Nakamura S. (2002). Tyrosine 125 of *α*-synuclein plays a critical role for dimerization following nitrative stress. Brain Res..

[48] Olivares D., Huang X., Branden L., Greig N.H., Rogers J.T. (2009). Physiological and pathological role of alpha-synuclein in parkinson’s disease through iron mediated oxidative stress; the role of a putative iron-responsive element. Int. J. Mol. Sci..

[49] Giasson B.I., Duda J.E., Murray I.V.J., Chen Q., Souza J.M., Hurtig H.I., Ischiropoulos H., Trojanowski J.Q.-Y., Lee V.M. (2000). Oxidative damage linked to neurodegeneration by selective *α*-synuclein nitration in synucleinopathy lesions. Science.

[50] Hodara R., Norris E.H., Giasson B.I., Mishizen-Eberz A.J., Lynch D.R., Lee V.M., Ischiropoulos H. (2004). Functional consequences of *α*-synuclein tyrosine nitration: Diminished binding to lipid vesicles and increased fibril formation. J. Biol. Chem..

[51] Hanahan D. (1983). Studies on transformation of *Escherichia coli* with plasmids. J. Mol. Biol..

[52] Croucher D.R., Iconomou M., Hastings J.F., Kennedy S.P., Han J.Z., Shearer R.F., McKenna J., Wan A., Lau J., Aparicio S. (2016). Bimolecular complementation affinity purification (BiCAP) reveals dimer-specific protein interactions for ERBB2 dimers. Sci. Signal..

[53] Derkatch I.L., Bradley M., Zhou P., Chernoff Y.O., Liebman S.W. (1997). Genetic and environmental factors affecting the de novo appearance of the [*PSI*^+^] prion in *Saccharomyces cerevisiae*. Genetics.

[54] Gietz R.D., Woods R.A. (2002). Transformation of yeast by lithium acetate/single-stranded carrier DNA/polyethylene glycol method. Methods Enzymol..

[55] Kaiser C., Michaelis S., Mitchell A. (1994). Methods in Yeast Genetics.

[56] Studier F.W., Moffatt B.A. (1986). Use of bacteriophage T7 RNA polymerase to direct selective high-level expression of cloned genes. J. Mol. Biol..

[57] Kushnirov V.V., Alexandrov I.M., Mitkevich O.V., Shkundina I.S., Ter-Avanesyan M.D. (2006). Purification and analysis of prion and amyloid aggregates. Methods.

[58] Drozdova P.B., Barbitoff Y.A., Belousov M.V., Skitchenko R.K., Rogoza T.M., Leclercq J.Y., Kajava A.V., Matveenko A.G., Zhouravleva G.A., Bondarev S.A. (2020). Estimation of amyloid aggregate sizes with semi-denaturing detergent agarose gel electrophoresis and its limitations. Prion.

