# Supplementary material for: NOS1AP Interacts with α-Synuclein and Aggregates in Yeast and Mammalian Cells
Source: Int J Mol Sci. 2022 Aug 14;23(16):9102. doi: 10.3390/ijms23169102 (PMC9409085; doi:10.3390/ijms23169102)
Supplement: Supplementary file 1 [file ijms-23-09102-s001.zip › ijms-1841598-supplementary.pdf]

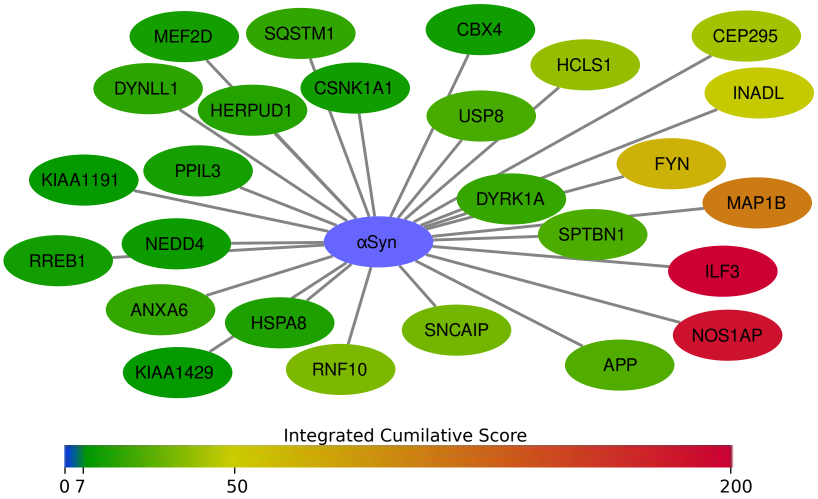

Figure S1. Network of potentially amyloidogenic proteins physically interacting with α-synuclein (αSyn). Node color corresponds to the Integrated Cumulative Score for a protein, calculated with ArchCandy. The ArchCandy Cumulative Score reflects the ability of the protein to form β-arches.
